# Supercritical CO_2_ extraction of artemisinin from *Artemisia annua* plant and the biotechnological production of artemisinin precursors: A dual-focus review

**DOI:** 10.1016/j.biotno.2025.05.003

**Published:** 2025-05-26

**Authors:** Babatunde Oladipo, Tunde V. Ojumu

**Affiliations:** Department of Chemical Engineering, Cape Peninsula University of Technology, Bellville, Cape Town, 7535, South Africa

**Keywords:** Artemisinin, Extraction, *Artemisia annua* L., Supercritical CO_2_, Microbial fermentation, Artemisinic acid, Amorphadiene

## Abstract

Artemisinin, a vital compound renowned for its potent antimalarial properties, has garnered significant attention due to its therapeutic importance and critical role in combating malaria. The extraction process is essential in recovering artemisinin from *Artemisia annua* L. plant. Supercritical carbon dioxide (scCO_2_) extraction has emerged as a highly effective and eco-friendly technique, offering improved efficiency, selectivity, and greener processing than conventional solvent-based methods. Despite this advancement, plant-derived artemisinin faces challenges in meeting global demand due to naturally low yields, seasonal variation, and agricultural limitations. Biotechnological advances have enabled the microbial production of artemisinin precursors, such as artemisinic acid and amorphadiene, which can be chemically or enzymatically converted into artemisinin, providing a scalable and sustainable production route. Despite the significance of both approaches, existing literature often treats them in isolation. Therefore, this work provides a comprehensive review, integrating scCO_2_ extraction technologies with microbial-based fermentation strategies for producing artemisinin and its precursors. Key parameters influencing scCO_2_ extraction efficiency, such as CO_2_ flow rate, temperature, co-solvent use, and pressure, are analyzed alongside fermentation bioprocess factors such as strain selection, pH, dissolved oxygen levels, carbon sources, and fermentation modes. By evaluating these complementary strategies, this review provides a holistic perspective aimed at improving artemisinin production yield, for accessibility and sustainability, ensuring a reliable global supply. It concludes by highlighting current challenges and proposing future directions necessary for optimizing the integrated production pipeline of artemisinin and its precursors.

## Highlights

• Antimalarial properties of artemisinin drive interest in its sustainable production.
• ScCO_2_ is efficient and eco-friendly for artemisinin extraction from *Artemisia annua.*
• Microbial synthesis offers scalable alternatives to meet artemisinin demand.
• Review explores integrating scCO_2_ extraction with fermentation for higher yield.
• Challenges and prospects for sustainable artemisinin production are addressed.

CYP71AV1: cytochrome P450 monooxygenase
DMAPP: dimethylallyl pyrophosphate
DO: dissolved oxygen
FPP: farnesyl pyrophosphate
HMG-CoA: 3-hydroxy-3-methylglutaryl-CoA
IPP: isopentenyl pyrophosphate
MEP: methylerythritol phosphate
MVA: mevalonate
ROS: reactive oxygen species
RSM: response surface methodology
SCFs: supercritical fluids
scCO_2_: supercritical carbon dioxide
SFE: supercritical fluid extraction
WHO: World Health Organization

## 1 Introduction

**Fig. 1:**
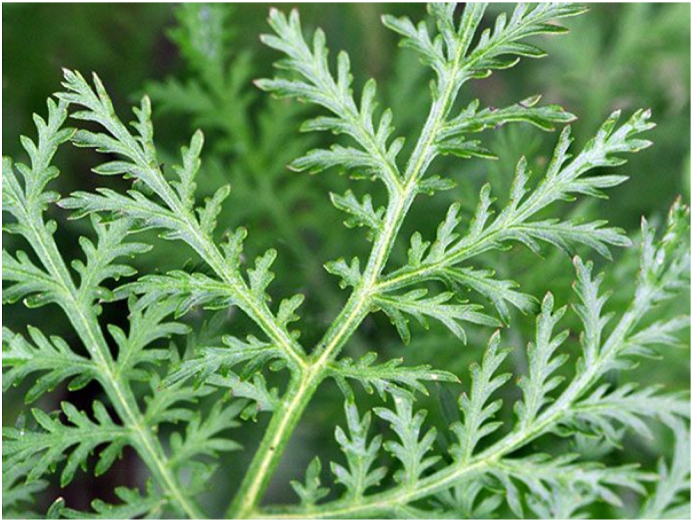
Sweet wormwood plant (*Artemisia annua*).

Despite advancements in malaria control efforts, such as routine immunizations, the number of cases in 2023 still exceeded that of 2022 by ∼11 million. In 2023, there were around 263 million incidents of malaria and 597,000 malaria-related deaths across 83 malaria-endemic countries.^1^ Artemisinin, a sesquiterpene lactone, is a pivotal natural compound in modern medicine due to its potent antimalarial properties. Its discovery and subsequent utilization significantly improved the fight against malaria, a disease threatening millions of people, especially in developing countries. The origin of artemisinin is traced back to traditional Chinese medicine and the discovery efforts in the 1970s by scientist Tu Youyou and her team.^2^ This groundbreaking discovery earned her the Nobel Prize in Physiology or Medicine in 2015.^3^ Artemisinin is primarily found in sweet wormwood plant (*Artemisia annua* L.), also commonly called qinghao. The plant (Fig. 1) is cultivated in various regions around the globe, particularly in Asia and Africa.^4^ Although there are up to 15 *Artemisia* species, artemisinin has the highest concentration in *A. annua* plant.^5^ Its concentration in the plant varies with factors such as geographical location, climate, and plant maturity.^6^ Typical artemisinin in *A. annua* has variations in concentration in various parts of the plant in the following order: leaves > flowers > stems > roots.^5^

The constituents of *A. annua* plant include artemisinin and its precursors like artemisinic acid, amorpha-4,11-diene, and dihydroartemisinic acid, along with a range of different bioactive compounds. These include flavonoids such as quercetin, luteolin, and kaempferol glycosides; terpenoids like artemisitene, camphor, and α-pinene; and essential oils containing 1,8-cineole and other volatile compounds.^7^^,^^8^ Additionally, the plant contains phenolic acids such as chlorogenic acid and caffeic acid, coumarins, sterols, and saponins.^9^ These constituents collectively contribute to the diverse pharmacological characteristics of the plant, such as anti-inflammatory, anticancer, antimicrobial, antioxidant, and antimalarial impacts.^10^^,^^11^ However, artemisinin is present in high concentrations and is the primary compound of pharmacological interest.

## 2 Artemisinin and its precursors: extraction and microbial production strategy

**Fig. 2:**
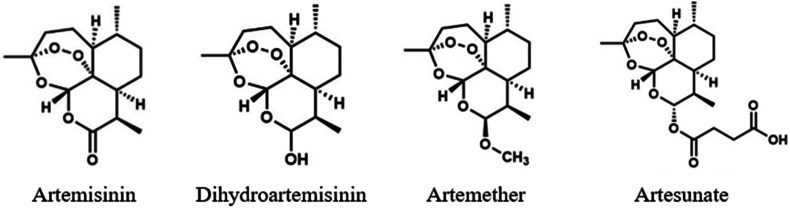
Structure of artemisinin and its derivatives^26^.

Artemisinin, along with its derivatives like dihydroartemisinin, artemether, and artesunate (Fig. 2), constitute essential elements in artemisinin-based combination therapies (ACTs), which the World Health Organization (WHO) endorses as the primary treatment protocols for malaria.^12^^,^^13^ The growing resistance of *Plasmodium falciparum* (the most deadly malaria parasite) to traditional monotherapies such as chloroquine and sulfadoxine-pyrimethamine highlights the critical importance of ACTs in malaria treatment. ACTs have demonstrated high efficacy against malaria parasites and remain the cornerstone of global antimalarial strategies.^14^^,^^15^ Thus, this has made the demand for ACTs substantial and continues to rise due to the persistent burden of malaria in the world. According to the ACT market size report (Grand View Research), it was reported that the global ACT market in 2023 was valued at 597.2 million USD and is expected to expand at a compound annual growth rate of 8.2 % from 2024 to 2030. This projected growth reflects the increasing prevalence of malaria and a need for a consistent supply of readily available and cost-effective therapeutic solutions.

Artemisinin has shown efficacy not only against malaria but also against various other parasitic infections, such as *Leishmania,*^16^ *Schistosoma*,^17^ *Toxoplasma*,^18^ and *Trypanosoma*^19^ species. In addition to its antiparasitic effects, it exhibits antiviral properties^20^ and has been investigated for its potential in treating hepatitis B.^21^ Moreover, artemisinin and its derivatives have demonstrated cytotoxic activity against multiple cancer cell lines, such as those associated with breast cancer, leukemia, colon cancer, and small-cell lung carcinoma.^22^^,^^23^ Notably, it may offer therapeutic benefits in managing drug-resistant cancers.^24^^,^^25^

The molecular structure of artemisinin comprises a unique endoperoxide bridge, essential for its antimalarial activity. When activated by iron, this bridge generates free radicals and oxygen species that can react, which induces oxidative stress, thereby causing damage to the cellular components of the parasite, such as the proteins and lipids. The resulting oxidative stress ultimately leads to the death of the malaria parasite.^27^, ^28^, ^29^ Despite its remarkable therapeutic potential and an annual pharmaceutical output exceeding 100 million treatments,^30^ artemisinin poses challenges in drug formulation due to its limited solubility. It dissolves moderately in polar organic solvents such as ethanol and methanol, while its solubility in water is notably poor, complicating both its extraction and formulation processes.^31^, ^32^, ^33^ This solubility characteristic behaviour of artemisinin influences the selection of appropriate extraction methods to yield high-purity artemisinin extracts efficiently.

The extraction process stands as the primary and crucial phase in the industrial production of artemisinin from *A. annua*. Conventional methods such as maceration,^34^^,^^35^ pressurized hot water extraction,^36^ and solvent-based solid-liquid extraction^37^^,^^38^ represent the commonly employed techniques to isolate artemisinin from *A. annua*. Various organic solvents like hexane, ethanol, toluene, ethyl acetate, acetone, chloroform, isopropanol, etc, have been documented in the literature.^39^, ^40^, ^41^ The utilization of solvents for the extraction process has given rise to various challenges, such as in the purity, activity, and stability of the artemisinin compound. For instance, artemisinin has low solubility in hexane, as well as the potential for rapid degradation in hot conditions, potentially influencing both the quantity and quality of the extracted product.^41^ Ethyl acetate has been identified for its ability to solubilize co-metabolites,^31^ while ethanol extraction yields a less pure extract by facilitating the simultaneous extraction of additional plant components.^42^ All these concerns complicate the purification steps for the recovery of artemisinin, thereby raising the overall cost of production. Moreover, the potential hazards to health and the environment, along with the associated disposal costs of organic solvents used in the process, have also added to the general drawbacks of their use. In addition, the practical application of conventional techniques faces limitations due to their substantial solvent consumption, slow extraction rates, and high energy requirements.^43^ In light of these limitations, researchers are actively exploring innovative, efficient, cost-effective, eco-friendly, and safe extraction methods to extract artemisinin on a large scale without compromising its inherent characteristics.

Literature indicates the emergence of efficient techniques for the extraction of artemisinin, such as supercritical fluid extraction (SFE),^44^^,^^45^ microwave-assisted extraction,^46^ and ultrasonic-assisted extraction.^47^^,^^48^ SFE technology using scCO_2_ is regarded as an eco-friendly technique since it eliminates the need for toxic solvents and mitigates thermal degradation by operating at lower temperatures. This technique facilitates continuous extraction procedures without the need for significant heat requirements to evaporate solvents.^37^^,^^42^ Furthermore, SFE facilitates selective extraction by the simple adjustment of operational variables such as flow rate, temperature, and pressure. This versatility allows the technology to be suitable for extracting various bioactive compounds without necessitating a change of the extraction solvent. Table 1 provides a summary of the merits and demerits of the technologies used for artemisinin extraction from *A. annua* plant.

**Table 1:** Merits and demerits of the extraction methods of artemisinin^37^^,^^44^, ^45^, ^46^, ^47^, ^48^.

Extraction method		Merits		Demerits
Soxhlet extraction		• The process is easy to set up, requiring less sophisticated equipment • Cost-effective process • Consistent and reproducible extraction process • Allows for prolonged contact between the solvent and the plant material on a continuous cycle		• Time-consuming process, as it typically requires several hours to complete a single extraction cycle • It requires a large volume of solvent to achieve efficient extraction • Thermal degradation of artemisinin may occur due to continuous heating and refluxing • Not a selective process, as there is the possibility of extracting other unwanted compounds, leading to impurities in the final product • The technique is not easily scalable for large-scale production • Pose health risks due to the toxicity of the solvents involved
Microwave-assisted		• Lower solvent consumption • Shorter extraction times • Higher rate of extraction • Minimizes energy loss		• Non-uniform heating of the plant material may lead to uneven extraction • Intense microwave energy may cause decomposition of the target compound • Potential safety risks due to the generation of heat • Limited scalability to large-scale extraction
Ultrasound-assisted		• Reduction in solvent consumption • Shorter operating times • Enhanced extraction efficiency • Low energy consumption		• Cavitation of bubbles in the solvent may lead to plant cell damage and affect the extract quality • Not compatible with all solvents. Some solvents may absorb ultrasonic waves differently, which can affect the extraction • Risk of human exposure to high-intensity ultrasonic waves can damage biological tissues • Generation of localized heat can degrade the target compound • Scalability to industrial levels may be challenging
Supercritical CO_2_ extraction		• More efficient as it offers a higher extraction rate in a shorter time frame. • High selectivity to extract target compounds • Preservation of the bioactivity of the extract due to mild operating temperature (31.1 °C) • High purity process as it leaves no residual solvent in the final extract • CO_2_ is considered to be non-toxic, non-flammable, and generally recognized as safe (GRAS) • Adjustable solvating power of scCO_2_ by adjusting pressure and temperature, allowing for process optimization • Often requires less energy, contributing to lower operational costs and a reduced environmental footprint. • Can be integrated with analytical chromatographic techniques such as gas chromatography (GC)		• The use of high pressures can contribute to high overall plant and operational costs • Phase equilibrium of the solvent/solute system is complex, which poses difficulties when designing extraction conditions • Addition of co-solvents or modifiers to enhance compound solubility may add complexity to the process • Typically expensive equipment

Despite its effectiveness as a frontline treatment for malaria, artemisinin derived from *A. annua* plant faces major limitations in meeting global demand. Following the WHO recommendation of artemisinin-based therapies, the availability and price of artemisinin fluctuated greatly, ranging from shortages in some years to excess supply in others. This inconsistency stems from the inherently low yields of the plant and high production costs. These limitations, compounded by environmental and geopolitical disruptions in the supply chain, continue to hinder the stable and sustainable production of artemisinin-based treatments.^49^^,^^50^

To address the challenges of limited supply and fluctuating prices of plant-derived artemisinin, researchers have explored alternative production methods. One promising advancement is the use of genetically engineered microbial systems, particularly yeast, to produce artemisinin precursors, such as artemisinic acid and amorphadiene, via microbial fermentation. These intermediates are subsequently isolated from the fermentation medium and then converted into artemisinin through chemical or enzymatic processes in a semi-synthetic pathway.^51^, ^52^, ^53^, ^54^ This breakthrough was made possible by identifying and incorporating *A. annua* genes responsible for artemisinic acid biosynthesis into yeast, alongside extensive metabolic engineering using synthetic biology tools. This approach led to the commercial-scale production of semi-synthetic artemisinin in 2013. Recent research continues to refine both the biological aspects of artemisinin biosynthesis in *A. annua* and microbial host optimization, as well as improving the downstream chemical conversion of artemisinic acid into artemisinin.^55^, ^56^, ^57^ These biotechnological and semi-synthetic strategies provide scalable, reliable, and more sustainable solutions for meeting global demand for this essential antimalarial drug.

The existing reviews on artemisinin extraction have focused on the comparative assessment of its extraction technologies and advances in its analytical quantification.^37^^,^^38^ However, we believe that the growing attention given to supercritical extraction of artemisinin warrants a detailed review, especially focusing on key process parameters that influence extraction efficiency, an area that remains underexplored in the existing literature. From the biotechnological point of view, recent developments for the commercial production of semi-synthetic artemisinin,^55^ artemisinin production strategies at an industrial scale,^58^ and biotechnological artemisinin production in plants,^59^ have been reviewed in the literature. However, the critical factors influencing the microbial production of artemisinin precursors remain underexplored. This review addresses that gap by examining key challenges and laying the foundation for developing terpenoid production directly from sugars. It also highlights future research directions to enhance the artemisinin production pipeline, such as integrating scCO_2_ technology for isolating artemisinin precursors from microbial fermentation.

## 3 Principles of supercritical fluid extraction technology

**Fig. 3:**
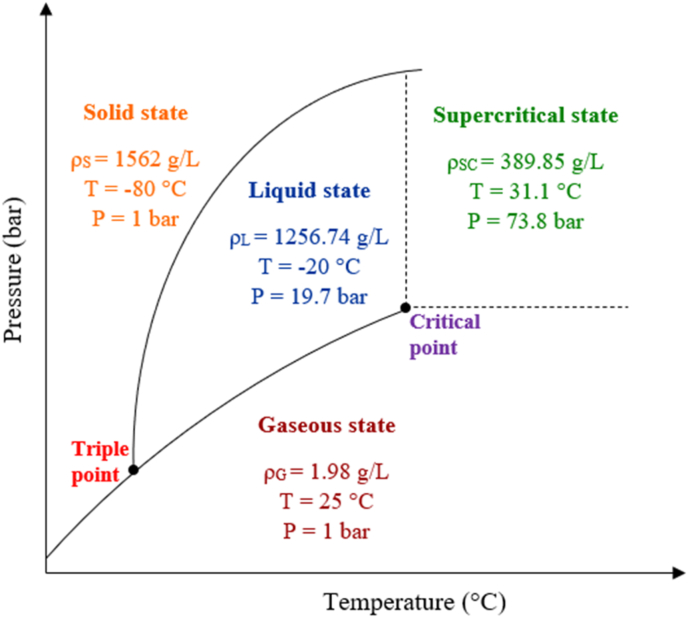
A schematic CO_2_ phase diagram showing the various states (modified from Qamar et al.^64^).

Supercritical fluids (SCFs) are substances that exist under conditions of temperature and pressure that exceed their critical points, thereby exhibiting a combination of qualities from both liquids and gases. Under these conditions, the substance exists as a single phase with unique solvent properties characterized by high diffusivity and low viscosity.^60^, ^61^, ^62^ The critical point is defined by specific values of temperature and pressure beyond which the distinction between liquid and gas phases ceases to exist under normal conditions, leading to the formation of a supercritical fluid. SCFs have been extensively utilized in SFE due to their high solvent power and solute selectivity to solubilize a wide range of compounds while offering the advantage of being eco-friendly.^60^^,^^61^^,^^63^ Presently, CO_2_ stands as the primary solvent choice in most SFE technologies because of its non-toxic, inert, non-flammable, selectively solvating, and non-polluting attributes. scCO_2_ also exhibits excellent wettability and diffusion capabilities owing to its inherently low surface tension.^60^^,^^64^ Moreover, employing scCO_2_ enables the alteration of the extract composition by adjusting pertinent process factors such as pressure, temperature, and flow rate. This flexibility allows the extraction of both volatile and non-volatile compounds. The critical zone for CO_2_ occurs at 31.1 °C and 73.8 bar (Fig. 3). Precisely, scCO_2_ exhibits gas-like diffusivity, high density akin to a liquid, and viscosity resembling a combination of gas and liquid. In this supercritical state, CO_2_ becomes an excellent solvent, capable of penetrating solids like a gas but dissolving compounds like a liquid. The gas-like diffusion properties and liquid-like solvating abilities of scCO_2_ make it suitable for selective and efficient extractions across different industries. Typically, the solvating capability of solvents is assessed based on their density. During the SFE process, CO_2_ initially permeates the entire plant matrix, leveraging its solvent density traits to dissolve valuable phytochemicals.^64^^,^^65^

**Fig. 4:**
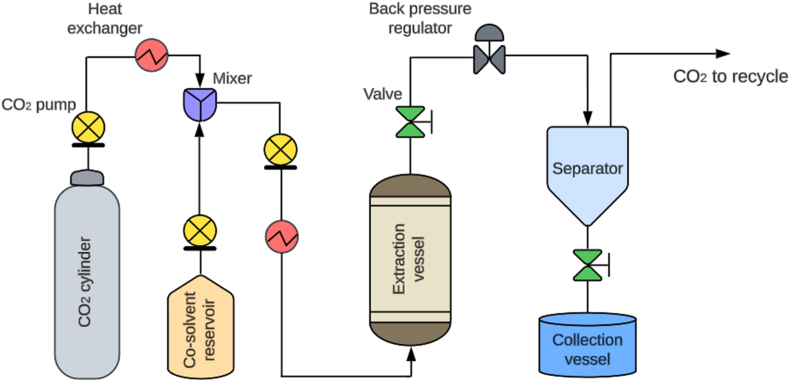
A schematic representation of the SFE system.

The diagram illustrating a laboratory-scale SFE system is shown in Fig. 4. In this system, liquefied CO_2_ stored in a cylinder is directed through a high-pressure pump. The liquid CO_2_ undergoes compression to achieve the appropriate pressure while concurrently being heated to attain the required extraction temperature, thereby reaching supercritical conditions. In some SFE systems, a specified amount of co-solvent like water, ethanol, or methanol may be injected into the SCF stream to enhance its selectivity and solvation properties.^44^

The scCO_2_ subsequently flows into the extraction vessel, where it performs the extraction on the plant material inside it. The scCO_2_ carrying the dissolved compounds flows through a depressurization valve, where the pressure is lowered upon exiting the extractor. This reduction in pressure causes CO_2_ to return to a gaseous state, leaving the extracted compounds behind. These extracted compounds are collected separately after the CO_2_ has been separated, leaving a pure extract without solvent residues. The gaseous CO_2_ can be captured, recompressed, and recycled back into the system for reuse, promoting an environmentally sustainable extraction process.^66^^,^^67^ This form of SFE is classified as off-line SFE as it involves conducting the extraction process separately from the main production line. It is usually employed when the extraction volume is relatively small compared to the main production output. On the other hand, online SFE is integrated directly into the production line where the material is being processed. The extraction process occurs continuously or intermittently and is advantageous for industries requiring immediate extraction of components from feed materials without interrupting the production flow.^68^ Occasionally, online SFE is employed for analytical functions where it is connected with analytical equipment like high-performance liquid chromatography, enabling the separation of substances in the extract into different components.^68^^,^^69^ Similarly, SFE can be applied in two distinct modes: dynamic and static. In the dynamic mode, the SCF continuously flows through the extraction vessel, carrying away the dissolved solutes as it passes through the feed material. The dynamic SFE mode is suitable for continuous processing and larger-scale operations. In contrast, the static mode involves placing the feed material in a vessel with SCF at a constant pressure and temperature. The solvent remains stationary, allowing it to permeate the material and dissolve the desired compounds. Static SFE mode is advantageous for smaller-scale operations and when precise control over extraction conditions is needed.^70^

## 4 Factors affecting artemisinin extraction efficiency in SFE technology

scCO_2_ is an effective solvent for extracting artemisinin from *A. annua* due to the non-polar nature of both the compound and the solvent. This method has been successfully applied to various plant parts, such as the leaves,^42^^,^^44^^,^^71^ aerial sections,^45^^,^^72^ and even the whole plant,^73^^,^^74^ to get the maximum extract possible. The selection of working conditions in SFE is contingent upon the specific compounds intended for extraction. Nevertheless, several critical factors must be carefully considered to ensure an effective extraction process, such as temperature, pressure, sample particle size, solvent flow rate, processing time, and co-solvent concentration.

### 4.1 Influence of temperature

Temperature regulation in SFE is commonly managed through a thermostatic bath or chamber in laboratory-scale setups, while industrial processes often employ concentric fluid heat exchange tubing for this purpose. The precise control of temperature holds significant importance in the extraction of artemisinin, given its sensitivity to temperature fluctuations. Nevertheless, the influence of temperature is contingent on the applied pressure and does not consistently exhibit a straightforward correlation with SFE efficiency. This is attributed to the interplay of two key variables: density and vapor pressure, which govern the dual and opposing effects of temperature during SFE. Under constant pressure, increasing temperature decreases the density of scCO_2_, thereby reducing its solvating power and consequently reducing the solubility of the compound.^75^ Conversely, at constant pressure, increasing temperature elevates the vapor pressure of the targeted compounds, thereby enhancing their solubility and extraction yield.^76^ These contrasting effects can lead to retrogradation, a term that describes a phenomenon where the solubility of compounds no longer correlates directly with changes in fluid density. This phenomenon can cause isotherms (lines on a graph representing constant temperature) to cross over, leading to unexpected variations in extraction efficiency, making the relationship between temperature and solubility a complex and nonlinear type.^77^^,^^78^ Various researchers have offered insights into the crossover phenomenon observed in how temperature affects the solubility of artemisinin. In the work of Ciftci et al.^42^ on the extraction of artemisinin from *A. annua* L., the authors noted a crossover phenomenon that had a partial impact on the artemisinin yields. Their investigation revealed a decrease in artemisinin yield as temperature increased at a pressure of 14 MPa, while observing an increase in artemisinin yield with increasing pressure at 26 MPa. This phenomenon also prevailed in the solubility of crystalline artemisinin. Xing et al.^79^ quantified the solubility of artemisinin in scCO_2_. According to their findings, at pressures above 19 MPa, the vapor pressure of artemisinin emerges as the predominant factor, leading to increased solubility with higher temperatures. However, within the pressure range of 10–19 MPa, the solubility of artemisinin decreases as temperature increases due to the predominant influence of density. In another study, Gong and Cao^80^ observed that the influence of temperature increase on the solubility of artemisinin transitions from being negative at pressures below the crossover zone (20–23 MPa) to positive at pressures above the crossover zone. This indicates that increasing temperature induces a diminished interaction between CO_2_ and artemisinin solute molecules, leading to a reduction in the enhancing factor and subsequently causing a decrease in solubility. Therefore, extraction temperature impacts the extraction of artemisinin, and optimizing this crucial variable is essential to maximizing the extraction yield while maintaining the quality of the extract.

### 4.2 Effect of pressure

The operating pressure also contributes to the behaviour and performance of the solvent employed in an SFE process. Elevated pressure at a specific temperature in SFE increases the density of the solvent, which improves the solvating power of the fluid. This enhancement in fluid density improves molecular mobility and reduces the mean free path of molecules, facilitating greater dissolution of target compounds. Hence, the greater the pressure, the less the solvent volume required for an effective extraction.^78^ However, elevating the pressure above a particular threshold may decrease the diffusivity of the supercritical fluid, leading to a decrease in solute dissolution.^81^ Ciftci et al.^42^ reported that the solubility of artemisinin in scCO_2_ increases with pressure. Using pure scCO_2_, Quispe-Condori et al.^82^ investigated the global yield isotherms and kinetics of artemisinin extraction. The researchers noted a potential inversion pressure within the range of 200–250 bar. They observed that the change in artemisinin yield with pressure resembled the global yield pattern at 50 °C, revealing a twelvefold increase in the extract obtained when the pressure was raised from 75 to 100 bar. Their results also indicated that at 30 °C, artemisinin yield remained relatively constant with pressure. However, at 50 °C, it was observed that a further increase in pressure positively influenced the extraction yield, but not to the same extent as observed at lower pressures. Excessively high pressures can lead to increased viscosity and reduced mass transfer rates, which may limit extraction efficiency. Pressure regulation in SFE can be achieved by utilizing a back pressure regulator (BPR) that ensures the CO_2_ pressure remains at the intended level.^83^

### 4.3 Influence of co-solvent/modifier

A liquid co-solvent refers to an organic solvent that exhibits the ability to dissolve in CO_2_ when combined in different proportions, thereby augmenting the solvent capability of the supercritical fluid towards specific targeted compounds.^84^ In SFE, co-solvents can be incorporated into the system in three distinct manners: blending them with the primary fluid in the pumping system, applying them as a co-solvent directly to the samples before extraction, and through the use of a cylinder tank containing pre-modified CO_2_. However, the latter technique is relatively costly and infrequently employed.^85^ Indeed, scCO_2_ serves as an efficient solvent for lipophilic (non-polar) compounds; however, its affinity for polar compounds is comparatively low.^75^^,^^77^ To address this issue, polar co-solvents or modifiers such as ethanol, methanol, water, and other polar agents can be incorporated during extraction to improve the solvating capabilities of scCO_2_, fostering better affinity for less soluble solutes, thereby enhancing solubility and ultimately increasing the extraction yield.^86^^,^^87^ The type and proportion of co-solvent selected play a crucial role in the extraction process, greatly affecting the solubility of the target compounds in SFE. Methanol and ethanol are frequently employed as co-solvents in extraction, typically at concentrations less than 10 % of the amount of CO_2_ used in the process.^78^^,^^88^ Ethanol is notably regarded as less toxic when compared to methanol. Numerous researchers have investigated the influence of co-solvents and their concentrations on scCO_2_ extraction, yielding significant implications. For example, Tzeng et al.^73^ found that adding 16.25 wt% ethanol to scCO_2_ extraction at 31.13 MPa and 40 °C resulted in the highest amount of artemisinin from *A. annua* L. plant, having 96 % recovery. In another study, Kohler et al.^89^ assessed the impact of various co-solvents/modifiers (methanol, methanol-water, ethanol, and toluene) on the extraction of artemisinin from *A. annua* L. using scCO_2_. The authors also tested different concentrations (1–10 %) of each modifier at operating conditions of 15 MPa, 50 °C, and a flow rate of 1 mL/min. The findings indicated that methanol, ethanol, and toluene gave a quantitative extraction, which was obtained in under 15 min with a 3 % modifier in CO_2_. However, toluene has a high boiling point, which leads to prolonged evaporation times, thus presenting a notable drawback. On the other hand, it was reported that only methanol-water yielded unsuccessful results, as evidenced by the extraction kinetics, which exhibited slower increases over an extended duration. This can be attributed to the low solvating capacity of water for artemisinin. In a more illustrative study using RSM, Ciftci et al.^42^ optimized the extraction process of artemisinin from *A. annua* L. with scCO_2_, where ethanol (0–12.6 wt%) was employed as a co-solvent. Their findings revealed that higher yields of artemisinin were achieved at lower co-solvent concentrations and elevated pressures and temperatures. Moreover, the authors reported that increasing the co-solvent concentration across all tested pressures and temperatures resulted in a decrease in artemisinin yield. This decrease was attributed to the incorporation of ethanol, which led to the extraction of pigments like chlorophyll, consequently lowering the artemisinin concentration in the extract. Lin et al.^90^ investigated the scCO_2_ and near-critical fluid extractions of artemisinin from *A. annua* plant under the processing conditions of 7–31.13 MPa and 30–60 °C. Their experimental results demonstrated that incrementally adding a co-solvent (such as n-hexane) effectively enhanced the recovery of artemisinin during extractions conducted near the critical region. Although an appropriate combination of pressure and temperature can enhance the solubility of artemisinin, adding a polar co-solvent may cause other polar molecules to be extracted as well, potentially compromising the selective extraction of artemisinin. Undesirable compounds, such as polyphenols, anthocyanins, and other colouring agents, have the potential to co-extract during the sCO_2_ processing of plant materials. In contrast to extracts obtained when ethanol was utilized as a co-solvent, Ciftci et al.^42^ observed that the extracts obtained without ethanol exhibited a lighter green colour. Furthermore, it was reported that the colour of the extract darkened with higher ethanol concentrations. Hence, increasing the ethanol concentration seems to enhance chlorophyll extraction, resulting in darker-coloured extracts and, consequently, reducing the purity of artemisinin extracts. The pharmaceutical industry places significant importance on the purity of extracts. A process that produces pure extracts eliminates the necessity for additional purification steps, thereby potentially reducing costs. An effective and successful SFE process should produce high-purity extracts with substantial yields. However, despite potential purity reductions, co-extracting other valuable compounds may offer additional advantages. Rodrigues et al.^44^ reported a similar phenomenon where the addition of ethanol to scCO_2_ increased the polarity of the solvent. Consequently, this enhancement improved its ability to dissolve other compounds present in the raw material in comparison to using pure scCO_2_. A comparable positive outcome was noted by Baldino et al.^45^ regarding the simultaneous extraction of essential oil when extracting antimalarial compounds from *A. annua* L. The authors highlighted the difficulty in separating *A. annua* essential oil from artemisinin due to their closely related compositions. However, the authors specified that because of their antioxidant properties, certain components of essential oils could be thought of as adjuvants to the mixture. This means they may contribute positively to the mixture by preventing oxidative damage and potentially enhancing the bioavailability or stability of artemisinin in the mixture. Therefore, it is crucial to evaluate each case individually, considering factors such as the desired product characteristics and economic feasibility.

### 4.4 Effect of CO_2_ density

The density of CO_2_ is a crucial parameter in artemisinin extraction as it determines the ability of the solvent to dissolve and transport the compound. Higher densities increase the solvating power, enabling efficient extraction of artemisinin. Studies have shown the impact of CO_2_ density and its flow rate on the extraction of artemisinin from *A. annua* plant. Tzeng et al.^73^ reported that the artemisinin content in the scCO_2_ extract increases with increasing fluid density. The appearance of the extracts obtained via SFE depends on the specific operational conditions employed during the extraction. For example, Quispe-Condori et al.^82^ observed that at lower solvent densities (50 °C/75 bar), the extract exhibited a lighter colour, whereas it became darker with increasing solvent density. Even at higher densities (30 °C/400 bar), the authors noted that the extract was lighter than that obtained through conventional solvent extraction. The darkening of the extract at higher solvent densities may be attributed to the extraction of higher molecular weight compounds. In another study on the optimization process for scCO_2_ extraction of antimalarial compounds from *A. annua*, Baldino et al.^45^ carried out an initial series of tests at 80 bar and 55 °C, with a CO_2_ density of 0.203 g/cm^3^. They observed that the active compounds received in the second separator were minimal, even after 400 min of processing. This was attributed to the low density of CO_2_ under the operating conditions, resulting in reduced solvent power towards artemisinin and its derivatives. An overall quasi-asymptotic yield was observed when CO_2_ density was raised to 0.288 g/cm^3^ at 90 bar and 50 °C, with fractional extraction incorporated into the process. When the operating conditions were adjusted to 100 bar and 40 °C, with a higher CO_2_ density of 0.623 g/cm^3^, a higher yield of active compounds and reduced co-extraction of high molecular weight compounds were achieved. The authors recovered a light-yellow liquid extract enriched in artemisinin in the second separator under conditions of 40 °C/100 bar, and recovered solid waxes in the first separator. Thus, the CO_2_ parameter is essential for optimizing yield while preserving the stability of artemisinin, making it a key focus in designing efficient and sustainable extraction processes.

### 4.5 Effect of CO_2_ or solvent flow rate

The rate at which the SCF flows through the plant matrix can significantly impact the extraction yield. The CO_2_ flow rate governs the mass transfer dynamics between the solvent and plant matrix. An optimal flow rate ensures sufficient contact time for effective solubilization of artemisinin while preventing solvent saturation, directly influencing extraction efficiency, yield, and process scalability.^91^ As the flow rate of sCO_2_ increases, the resistance to mass transfer decreases, thereby enhancing the extraction yield. Flow rates that are excessively high or low can lead to reduced extraction yields. Controlling the sCO_2_ flow rate is achieved through the use of a back-pressure regulator in conjunction with a gas flow meter. Adjustment of the flow rate is facilitated by employing a restrictor with different internal diameters.^66^ Conducting SFE studies at various solvent flow rates can provide insights into the mass transfer resistance experienced by scCO_2_ during the extraction process. In the study by Baldino et al.,^45^ the authors noted that increasing the scCO_2_ flow rate from 0.8 to 1.2 kg/h under the same process conditions had minimal impact on the extraction kinetics of different compounds. This suggests that the extraction process is primarily governed by internal mass transfer resistance. Kohler et al.^89^ examined the impact of CO_2_ flow rate (0.5–3 mL/min: liquid CO_2_ and modifier) during SFE of artemisinin from *A. annua* at operating conditions of 15 MPa, 50 °C, with 3 % methanol as a modifier. A favourable extraction of artemisinin was achieved in under 10 min at a 2 mL/min flow rate. Summarizing previous studies on scCO_2_, it was found that when SFE was conducted at low CO_2_ densities, the yield of artemisinin was very low. On the other hand, Lin et al.^90^ observed that high CO_2_ densities during SFE led to substantial co-extraction of unwanted compounds. Few studies have systematically varied the CO_2_ flow rate to specifically investigate its effect on artemisinin yield,^45^^,^^89^ leaving a gap in understanding how this parameter influences extraction efficiency. Further research is needed to explore how variation in CO_2_ or total solvent flow rate impacts solubilization, mass transfer, and overall extraction efficiency of artemisinin.

### 4.6 Influence of extraction time

The duration of operation is a crucial factor in determining the estimated production cost since it directly impacts the number of extraction cycles the unit can complete within a given timeframe.^92^ The duration of extraction is a key variable in SFE because it has a direct influence on the composition of the extract. Short extraction times may lead to incomplete extraction, whereas excessively long durations could result in a waste of time and solvent, along with the potential degradation of bioactive compounds. The extraction duration is contingent upon the flow rate; higher flow rates generally correspond to shorter extraction times.^78^^,^^85^ Conducting an initial study aimed at establishing the ideal time and fluid flow rate to achieve the maximum yield of extract is advisable. Ciftci et al.^42^ studied the impact of scCO_2_ extraction time by increasing the processing period from an initial 2.5–6 h under optimized conditions. Artemisinin yields of 0.71 % and 0.78 % were reported for extraction times of 2.5 and 6 h, respectively. Extending the extraction time did not significantly enhance either the yield of artemisinin or the purity of the extracts. Table 2 presents a summary of literature findings on the influence of *A. annua* plant matrix and SFE operating parameters on artemisinin yield.

**Table 2:** Application of scCO_2_ for the extraction of artemisinin from *Artemisia annua* L plant

*A. annua* matrix		SFE operating parameters		Optimal process conditions		Artemisinin yield or recovery		Analytical method		References
Whole plant		Pressure: 30 MPa Temperature: 40 °C Extraction time: 180 min CO_2_ flow rate: 1.4 kg/h		Pressure: 30 MPa Temperature: 40 °C Extraction time: 180 min CO_2_ flow rate: 1.4 kg/h		3.21 mg/g		HPLC		Banožić et al.^74^
Whole plant		Pressure: 17.34–31.13 MPa Temperature: 40–60 °C Co-solvent: ethanol (7–16.25 %) Extraction time: 60–720 min		Pressure: 24.23 MPa Temperature: 40 °C Co-solvent: ethanol (16.25 %)		11.36 mg/g		GC		Tzeng et al.^73^
Whole plant		Pressure: 7–31.13 MPa Temperature: 30–60 °C Co-solvent: n-hexane (0–22.56 wt%) Extraction time: 90 min		Pressure: 18.72 MPa Temperature: 37 °C Co-solvent: n-hexane (16.25 wt%) Extraction time: 90 min		2.8 mg/g		GC-FID		Lin et al.^90^
Leaves		Pressure: 20, 25, and 30 MPa Temperature: 40, 50, and 60 °C Co-solvent: ethanol (0, 15, and 25 %) Extraction time: 60 min Total solvent flow rate: 2.0 g/min		Pressure: 20 MPa Temperature: 60 °C Co-solvent: ethanol (0 %) Extraction time: 60 min		8.0 mg/g		HPLC		Rodrigues et al.^44^
Leaves		Pressure: 9.9, 14, 20, 26, and 30 MPa Temperature: 33, 40, 50, 60, and 67 °C Co-solvent: ethanol (0, 2.5, 6.3, 10, and 12.6 %) Extraction time: 150 min CO_2_ flow rate: 2 L/min		Pressure: 30 MPa Temperature: 33 °C Co-solvent: ethanol (0 %) Extraction time: 150 min CO_2_ flow rate: 2 L/min		7.1 mg/g		HPLC		Ciftci et al.^42^
Leaves		Pressure: 40 MPa Temperature: 60 °C CO_2_ flow rate: 0.00004 kg/s		Pressure: 40 MPa Temperature: 60 °C CO_2_ flow rate: 0.00004 kg/s		5.47 mg/g		HPLC		Martinez-Correa et al.^71^
Leaves		Pressure: 7.5–40 MPa Temperature: 30 and 50 °C CO_2_ flow rate: 0.000068 kg/s		Pressure: 30 MPa Temperature: 50 °C CO_2_ flow rate: 0.000068 kg/s		7.0 mg/g		GC-FID		Quispe-Condori et al.^82^
Aerial parts		Pressure: 8, 9, 10, and 20 MPa Temperature: 40, 50, and 55 °C CO_2_ density: 0.203, 0.288, 0.623, and 0.784, g/cm^3^ CO_2_ flow rate: 0.8 and 1.2 kg/h Extraction time: 60 min		Pressure: 10 MPa Temperature: 40 °C CO_2_ flow rate: 0.8 kg/h Extraction time: 60 min		5.0 mg/g		GC-MS		Baldino et al.^45^
Aerial parts		Pressure: 30 MPa Temperature: 50 °C CO_2_ flow rate: 11 ± 3 kg/h		Pressure: 30 MPa Temperature: 50 °C CO_2_ flow rate: 11 ± 3 kg/h		5.0 mg/g		HPLC		Ivanovic et al.^72^
Aerial parts		Pressure: 15–30 MPa Temperature: 50 °C Co-solvent: methanol (1, 3, 5, and 10 %) Extraction time: 2.5–30 min Solvent flow rate: 0.5–3 mL/min		Pressure: 15 MPa Temperature: 50 °C Co-solvent: methanol (3 %) Extraction time: 20 min Solvent flow rate: 2 mL/min		6.0 mg/g		SFC-FID		Kohler et al.^89^
Aerial parts		Pressure: 15–30 MPa Temperature: 50 °C Co-solvent: toluene (1, 3, 5, and 10 %) Extraction time: 2.5–30 min Solvent flow rate: 1 mL/min		Pressure: 15 MPa Temperature: 50 °C Co-solvent: toluene (3 %) Extraction time: 20 min Solvent flow rate: 1 mL/min		6.5 mg/g		SFC-FID		Kohler et al.^89^

**Fig. 5:**
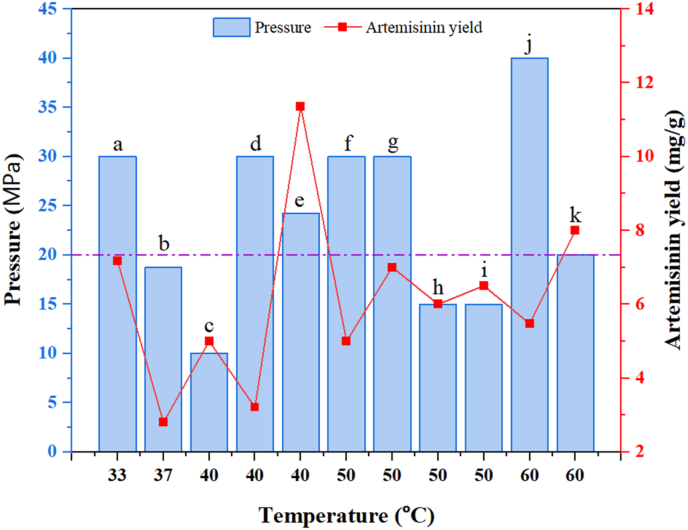
Effect of temperature and pressure on artemisinin yield (generated from Table 2).

From Tables 2 and it could be inferred that the highest yield of artemisinin was obtained in the study by Tzeng et al.^73^ when the whole plant (leaves, stems, aerial parts, flowers, and roots) was used as the feed material, coupled with the use of 16.25 wt% ethanol as a co-solvent. This suggests that the entire plant contains a more comprehensive range of compounds and reservoirs contributing to higher artemisinin extraction efficiency. Artemisinin is primarily located in trichomes, which are distributed across the whole plant.^95^ Utilizing the entire *A. annua* plant for extraction maximizes the number of trichomes available, leading to higher artemisinin yields. Further optimization could involve selectively harvesting part of the plant with the highest density of trichomes. The graphical representation of SFE pressure and temperature investigated in literature, as shown in Tables 2 and is presented in Fig. 5. We observed that the highest artemisinin yields were obtained in the temperature range of 40–50 °C, while the optimal pressure range for high yields is 15–20 MPa. Increasing the pressure beyond 20 MPa has no substantial effect on artemisinin yield. Artemisinin is relatively stable at temperatures below 50 °C, which is why most extraction processes aim to maintain the temperature within this range. Whereas it starts degrading significantly when the temperature exceeds 60 °C, leading to the breakdown of its peroxide bridge, which is crucial for its biological activity. scCO_2_ at pressures between 15 and 20 MPa enhances the density and solvating power of the solvent, improving the extraction efficiency without causing compound instability. From Fig. 5, it can be observed that there is no direct, linear correlation between temperature and pressure for maximum artemisinin yield.

a - Ciftci et al.,^42^ b - Lin et al.^90^ (Co-solvent: n-hexane (16.25 wt%)), c - Baldino et al.,^45^ d - Banožić et al.,^74^ e − Tzeng et al.^73^ (Co-solvent: ethanol (16.25 wt%)), f - Ivanovic et al.,^72^ g - Quispe-Condori et al.,^82^ h - Kohler et al.^89^ (Co-solvent: methanol (3 %)), i - Kohler et al.^89^ (Co-solvent: toluene (3 %)), j - Martinez-Correa et al.,^71^ k - Rodrigues et al.^44^

### 4.7 Influence of *Artemisia annua* raw matrix

Numerous factors, including the inherent characteristics of the source material, moisture levels, particle dimensions, porosity, and surface properties, play pivotal roles in affecting solubility and the mass transfer mechanism in scCO_2_ extraction.^93^ Table 3 shows the properties of *A. annua* plant. A careful selection and optimization of these factors can expedite the thorough extraction of desired compounds within a short duration.

**Table 3:** Properties of *A. annua* reported in the literature

Property	Martinez-Correa et al.^71^	Baldino et al.^45^	Tzeng et al.^73^	Quispe-Condori et al.^82^	Rodrigues et al.^44^
Plant source	Brazil	Italy	China	Brazil	Brazil
Moisture (%)	8.1 ± 0.1	12	9.7 ± 0.1	–	11 ± 0.5
Mean particle diameter (mm)	0.838	0.2	–	0.492	–
Real particle density (kg/m^3^)	1440 ± 0.04	–	–	950.9	–

It is customary to dry the sample material that will be used for extraction to reduce the amount of moisture present. This stage is crucial because any water in the sample could interfere with the solute and lower the extraction efficiency. However, in some cases, water must exist to promote desirable interactions between the solvent and the solute. The literature reports a moisture content range of 8–12 % for *A. annua* plant (Table 3). During the extraction process, the mass transfer rate is mostly determined by the porosity and particle size of the solid materials. Decreasing the particle size directly enhances the extraction process by shortening the diffusion traveling distance of the solvent and enlarging the area of contact, leading to an accelerated extraction process.^78^^,^^85^^,^^94^ Nevertheless, it is important to avoid using excessively fine particles, as they can increase resistance to internal mass transfer. This could potentially lead to column channeling, ultimately resulting in reduced process efficiency and lower yields in the extraction. Studies reported in the literature indicate that the mean particle size of *A. annua* plant utilized for SFE of artemisinin typically ranges between 0.2 and 0.8 mm (Table 3). However, at the time of writing this review, there is no available study that has investigated the influence of particle size during scCO_2_ extraction of artemisinin from *A. annua* plant.

We suggest that modeling and optimization tools such as Artificial Neural Networks (ANN) and Adaptive Neuro-Fuzzy Inference Systems (ANFIS) should be employed to critically examine the effects of SFE parameters on artemisinin yield due to the ability of these tools to model complex, nonlinear interactions between variables. These techniques allow for more precise identification of optimal conditions by considering the interdependence of factors such as temperature, pressure, CO_2_ flow rate, extraction time, and co-solvent concentration. Additionally, employing these approaches can reduce experimental costs and time by predicting outcomes with fewer trials. Incorporating real-time monitoring tools, such as in-line spectroscopy, can further provide insights into the dynamics of artemisinin extraction. These combined efforts will ensure a robust understanding of the extraction process and facilitate the determination of precise, scalable, and economically viable SFE parameters for efficient artemisinin recovery from *A. annua*.

## 5 Production of artemisinin precursor via fermentation and its purification

**Fig. 6:**
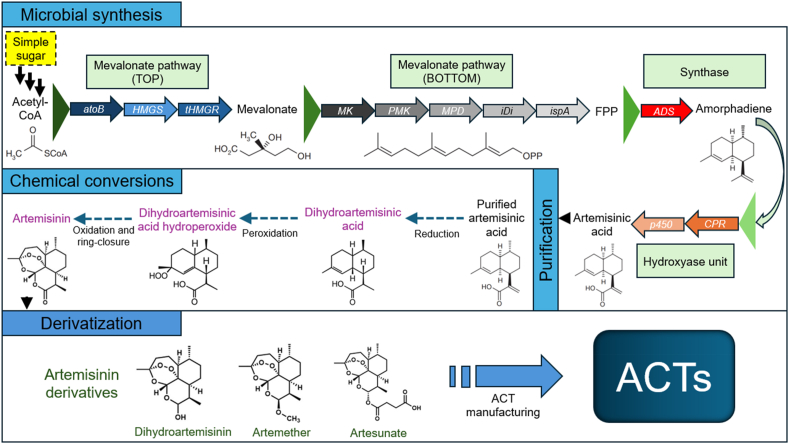
Engineered yeast microbial production of artemisinic acid and downstream processing of artemisinin and its derivatives (modified from Hale et al.^110^).

The global demand for malaria drugs cannot be met solely by *A. annua* cultivation due to its limited artemisinin yield, seasonal growth constraints, and dependence on agricultural and environmental conditions, which hinder scalability and consistent production.^49^^,^^50^ Moreover, artemisinin precursors, such as artemisinic acid, dihydroartemisinic acid, and amorphadiene, which are vital intermediates, cannot be effectively extracted using scCO_2_ because these precursors are typically present in very low concentrations in *A. annua*. A promising alternative approach involves the biosynthesis of artemisinic acid through microbial fermentation of the sesquiterpene intermediate, amorphadiene, which is subsequently converted into artemisinin via chemical transformation. This biotechnological approach offers a more sustainable, scalable, and reliable alternative for artemisinin production. In *A. annua* plant, artemisinin is naturally synthesized in glandular trichomes through the DOXP (1-deoxy-d-xylulose-5-phosphate) pathway, also called the MEP (methylerythritol phosphate) pathway. This plastidic pathway produces isoprenoid precursors, which are then converted into artemisinin through a series of intermediates such as amorpha-4,11-diene, artemisinic alcohol, artemisinic acid, and dihydroartemisinic acid. The final step of artemisinin formation involves non-enzymatic photooxidation and spontaneous chemical reactions.^95^^,^^96^ While the MEP pathway is naturally used by *A. annua*, it has some shortcomings, such as low flux and metabolic yield, complex regulation, plastid localization, and cofactor limitations. This has led researchers to favour the mevalonate (MVA) pathway. Engineered heterologous microbial fermentation systems, usually *Saccharomyces cerevisiae*^52^^,^^57^ and *Escherichia coli*^53^^,^^97^ utilize the MVA pathway to synthesize artemisinin precursors, such as artemisinic acid, from simple carbon sources like glucose. The artemisinic acid is then extracted and chemically converted into artemisinin (Fig. 6). While plant-based production relies on complex cellular structures and light-driven reactions, microbial systems enable controlled and more consistent production, though they require chemical steps to complete the synthesis.^96^ For instance, artemisinic acid production through fermentation begins with the selection of a microbial host, typically *S. cerevisiae* (yeast). In a controlled fermentation process, the engineered yeast is grown in bioreactors under optimized conditions. Carbon sources such as glucose provide the primary substrate, while the system environment is regulated for parameters like pH, temperature, and aeration to support high metabolic activity.^52^

During glycolysis, glucose is enzymatically converted into two molecules of pyruvate, with the concomitant production of adenosine triphosphate (ATP) and nicotinamide adenine dinucleotide (NADH).^55^^,^^98^ This is achieved via a series of enzymatic reactions such as those catalyzed by hexokinase, phosphofructokinase, and pyruvate kinase. Pyruvate then enters the mitochondrial or cytosolic metabolism, where it is converted into acetyl-CoA through the action of the pyruvate dehydrogenase complex, releasing CO_2_ in the process.^55^ Acetyl-CoA, a central metabolic intermediate, serves as the starting substrate for isoprenoid biosynthesis. In *S. cerevisiae*, the acetyl-CoA feeds into the MVA pathway, a native pathway in yeast but engineered for enhanced flux. First, two molecules of acetyl-CoA are condensed to form acetoacetyl-CoA by acetyl-CoA acetyltransferase. A third acetyl-CoA molecule is added by HMG-CoA synthase to yield 3-hydroxy-3-methylglutaryl-CoA (HMG-CoA). This compound is then reduced by the NADPH-dependent HMG-CoA reductase, an important rate-limiting step, to form mevalonic acid.^99^^,^^100^ Mevalonate is sequentially phosphorylated by mevalonate kinase and phosphomevalonate kinase and then decarboxylated by mevalonate diphosphate decarboxylase to produce isopentenyl pyrophosphate (IPP), a five-carbon isoprenoid unit. Some of the IPP is isomerized into dimethylallyl pyrophosphate (DMAPP) by IPP isomerase. These two C_5_ units (IPP and DMAPP) are condensed to form farnesyl pyrophosphate (FPP, C_15_) through the action of farnesyl pyrophosphate synthase.^51^^,^^99^

To divert FPP toward artemisinin precursor production rather than sterol biosynthesis, the engineered microbes express a plant-derived enzyme, amorpha-4,11-diene synthase (ADS), which catalyzes the cyclization of FPP to amorpha-4,11-diene.^51^ This sesquiterpene is then subjected to a series of oxidative modifications catalyzed by cytochrome P450 monooxygenase (CYP71AV1),^55^^,^^57^^,^^101^ a crucial enzyme sourced from *A. annua* and expressed in the microbial host. CYP71AV1 oxidizes amorpha-4,11-diene to artemisinic alcohol. This intermediate is further oxidized by alcohol dehydrogenase (ADH1) to artemisinic aldehyde, and then by aldehyde dehydrogenase (ALDH1) to form artemisinic acid, the final biosynthetic product of the microbial fermentation phase.^55^^,^^102^ The artemisinic acid is secreted into the culture medium.

Recovering artemisinic acid during fermentation poses challenges due to its cytotoxicity to host cells and high volatility, which can lead to product losses.^103^ To address this occurrence, product recovery techniques using solvent-based techniques such as liquid-liquid or solid-phase extraction are employed. These methods facilitate the isolation and subsequent purification of artemisinin precursors for downstream applications.^56^ However, solvent-based extraction methods often involve using organic solvents, which can pose environmental and safety risks and may leave residues in the final product. Additionally, these techniques can require high energy inputs for solvent recovery and may lead to the degradation of thermally sensitive compounds like artemisinic acid.^39^^,^^41^ Supercritical CO_2_ extraction offers a greener alternative for the recovery of fermentation-derived medical compounds, as it eliminates the need for harmful solvents, operates under mild conditions to preserve compound integrity, and allows precise control of the extraction process. Interest is increasing in utilizing supercritical fluid extraction to separate compounds derived from microbial fermentation processes.^104^^,^^105^ For example, acetoin^106^ and griseofulvin^107^ were successfully recovered using this technique.

By designing a continuous or semi-continuous interface between fermentation and scCO_2_ extraction, the produced artemisinic acid can be selectively partitioned into the scCO_2_ phase in real time or at regular intervals, thus enabling in situ product removal. Using scCO_2_ to remove the product in situ not only facilitates efficient recovery but also actively alleviates product accumulation within the bioreactor. This mitigation of intracellular and extracellular artemisinic acid concentrations significantly reduces its toxic effects on microbial metabolism, extending the productive phase of fermentation and potentially increasing overall titres. Furthermore, this integration reduces the need for complex solvent-based purification steps and minimizes contamination risks, since CO_2_ is inert and non-toxic to microbial cultures when applied under controlled pressure and temperature conditions. However, despite its advantages, scCO_2_ extraction has not been widely applied to recover artemisinin precursors after the fermentation process. This represents an untapped opportunity for enhancing sustainability and efficiency in the production of these critical compounds. Such an integrated bioprocess design demands careful optimization of parameters, such as scCO_2_ pressure, temperature, and flow rate, as well as the timing and configuration of the extraction interface to ensure compatibility with microbial growth conditions.

Since artemisinic acid is not itself active against malaria, it must undergo chemical conversion to yield artemisinin. This is accomplished through a semi-synthetic process involving two major steps. In the first step, artemisinic acid is hydrogenated to dihydroartemisinic acid using molecular hydrogen (H_2_) in the presence of a palladium-based catalyst (e.g., Pd/C).^55^ In the second step, dihydroartemisinic acid undergoes a photochemical oxidation in the presence of singlet oxygen (^1^O_2_),^55^ typically generated by light irradiation and a sensitizer such as methylene blue. This reaction leads to the formation of an unstable hydroperoxide intermediate, which spontaneously cyclizes to form artemisinin, a compound characterized by its endoperoxide bridge, a structural feature essential for its antimalarial activity.^51^^,^^56^ Artemisinin is then chemically derivatized to improve its pharmacological properties. It can be transformed into artesunate (via succinylation), artemether (via methylation), or dihydroartemisinin (via reduction).^108^^,^^109^ These derivatives are formulated into ACTs, and in combination with other antimalarial agents (e.g., lumefantrine, amodiaquine) ensure enhanced efficacy and help reduce the development of resistance.

## 6 Factors influencing the production of artemisinin precursors via fermentation

Several factors influence the production of artemisinin precursors in fermentation, ranging from the choice of host microorganism, pH, temperature, oxygen availability, and the composition of the fermentation medium, including carbon and nitrogen sources. This section offers a detailed examination of the important factors.

### 6.1 Effect of host microorganism

One of the most essential factors is the choice of the host microorganism. The eukaryotic cellular environment of *S. cerevisiae* supports robust growth, facilitates genetic manipulation, and efficiently utilizes glucose as a primary carbon source, making it a widely used host for the fermentation-based production of artemisinin precursors.^51^^,^^52^^,^^57^^,^^100^ Other microbes, such as *E. coli*, a prokaryotic host, have also been employed for their rapid growth and simpler genetic manipulation,^53^^,^^54^^,^^97^^,^^111^ but they face challenges in expressing eukaryotic enzymes, particularly cytochrome P450s. The genetic engineering of the host strain plays a pivotal role in optimizing the metabolic flux towards artemisinin precursor production. The metabolic robustness of the host organism also affects its ability to sustain high yields under industrial fermentation conditions. For example, *S. cerevisiae* tolerance to ethanol and other stressors, such as metabolic by-products like FPP and reactive oxygen species (ROS), enables prolonged production phases during fermentation,^52^^,^^57^ whereas *E. coli* may experience metabolic bottlenecks under similar conditions. Some important process parameters and titres obtained for artemisinin precursor biosynthesis are presented in Table 4.

**Table 4:** Artemisinin precursors produced via microbial fermentation

Microbial host	pH	T (°C)	Aeration, agitation	DO (%)	Operation mode	Precursor produced	Titre (g/L)	References
*S. cerevisiae*	5.0	30	1 L/min air	40	Fed-batch	Amorpha-4,11-diene	>40	Westfall et al.^52^
*S. cerevisiae*	5.0	30	1 L/min air	40	Fed-batch	Artemisinic acid	25	Paddon et al.^57^
*S. cerevisiae*	–	30	0.5 L/min air, 100–500 rpm	40	–	Artemisinic acid	0.1	Ro et al.^51^
*S. cerevisiae*	5.0	30	1 L/min, 300–1200 rpm	40	Fed-batch	Artemisinic acid	2.5	Lenihan et al.^100^
*S. cerevisiae*	5.5	37	250 rpm	–	–	Amorphadiene	0.497	Kwak et al.^98^
*E. coli*	7.0	30	1 vvm, 700 rpm	40	Fed-batch	Amorpha-4,11-diene	27.4	Tsuruta et al.^53^
*E. coli*	–	–	220 rpm	–	–	Amorpha-4,11-diene	0.293	Anthony et al.^54^
*E. coli*	7.0	30	1.5 vvm air, 800–2000 rpm	30	Fed-batch	Amorpha-4,11-diene	30	Shukal et al.^97^
*E. coli*	–	37	–	–	–	Amorpha-4,11-diene	0.1122	Martin et al.^111^

### 6.2 Influence of carbon source

The choice of carbon source in fermentation processes depends on the specific precursor pool targeted for biosynthesis. Ethanol has been frequently introduced into fermentation broths as a substrate because it is readily converted into acetyl-CoA, a critical intermediate for synthesis. This transformation positions ethanol as a more advantageous substrate compared to glucose during the production stage, resulting in greater titres and improved yields of sesquiterpenes in *S. cerevisiae*, such as amorpha-4,11-diene^52^ and artemisinic acid.^57^ These studies employed a diauxic fermentation approach, where glucose is metabolized in the initial growth phase, producing ethanol, which is subsequently utilized during the secondary phase for product biosynthesis. However, using ethanol as the primary carbon source is not economically viable for large-scale sesquiterpene production, as it is more expensive than alternatives like glucose. Consequently, glucose, which is the most commonly utilized carbon source in bioprocessing, has been adopted to achieve high titres and yields in the fermentation of sesquiterpene artemisinin precursors.^53^^,^^97^ Additionally, the selection of promoters for heterologous gene expression can influence substrate choice. For instance, when galactose-inducible promoters are used in engineered yeast strains, galactose must be included in the medium to induce gene expression while simultaneously serving as a carbon source. This approach increases production costs and poses regulatory challenges, as the expression driven by galactose is suppressed when glucose is present. To address this issue, the GAL1 gene was removed, allowing artemisinic acid synthesis to proceed with glucose as the main carbon source, supplemented with a small quantity of galactose as an inducer.^52^ Despite these adaptations, ethanol used in a diauxic yeast fermentation process has yielded the highest specific production rates among all other carbon sources.^52^^,^^57^

### 6.3 Effect of nitrogen source

Nitrogen plays a fundamental role in protein synthesis, enzymatic activities, and overall cellular metabolism. Common nitrogen sources used in the production of artemisinin precursors include yeast extract, ammonium salts (e.g., ammonium sulfate and ammonium chloride), and nitrates.^53^^,^^97^^,^^98^^,^^100^ These compounds are preferred due to their availability and compatibility with microbial cultures. When nitrogen sources are present in the fermentation medium, they are transported into cells via specific permeases and subsequently integrated into key metabolic pathways to support nitrogen assimilation and biosynthesis. The concentration and type of nitrogen source significantly influence the growth, metabolism, and gene expression of *S. cerevisiae.*^51^^,^^98^^,^^100^ While optimal nitrogen levels enhance cell growth and metabolite production, imbalances can negatively impact the process. For instance, high nitrogen concentrations may overstimulate yeast proliferation, leading to excessive biomass accumulation, depletion of other essential nutrients, and increased fermentation temperatures.^112^ These conditions can ultimately result in reduced production efficiency and fermentation failures. Conversely, nitrogen deficiency in prolonged fermentations can lead to slowed or halted metabolic activity, impairing the production of artemisinin precursors. The accumulation of toxic intermediates or ROS during artemisinic acid production has been linked to decreased cell viability under low nitrogen conditions.^57^ To address these challenges, strategies such as controlled and gradual nitrogen release during fermentation can prolong productivity, prevent sudden temperature spikes, and mitigate nutrient imbalances.^57^^,^^112^^,^^113^ In both shake-flask and bioreactor systems, optimizing the nitrogen source concentration is critical for maintaining microbial growth and enhancing sesquiterpene production.

### 6.4 Influence of temperature

The production of artemisinin precursors is sensitive to temperature, as it directly influences cellular metabolism and the activity of enzymes within the biosynthetic pathway. Optimal production is commonly reported at around 30 °C (Table 4). At high temperatures (>35 °C), key enzymes such as amorpha-4,11-diene synthase (ADS) and CYP71AV1 may undergo thermal denaturation or exhibit reduced catalytic efficiency, leading to metabolic imbalances, decreased precursor yields, and activation of heat shock and oxidative stress responses.^53^^,^^57^^,^^112^ On the other hand, temperatures below the optimal range (<25 °C) can slow down enzymatic reaction rates and microbial growth, thereby limiting flux through the MVA pathway responsible for precursor biosynthesis. Thus, maintaining an optimal and stable fermentation temperature is critical for maximizing the yield of artemisinin precursors.

### 6.5 Effect of pH

pH affects enzymatic activity, metabolic flux, and cell viability in microbial fermentation engineered systems. Literature indicates that the optimal pH for precursor production typically lies within a slightly acidic to neutral range (pH 5.0–7.0),^52^^,^^53^^,^^57^^,^^97^ depending on the microbial host and cultivation conditions. *S. cerevisiae*, commonly used in sesquiterpene production, performs well at pH ∼5, which supports growth and maintains the stability of acid-tolerant enzymes in the MVA pathway. In contrast, *E. coli* exhibits better growth and expression efficiency at pH ∼7 (Table 4), aligning with its native physiology and supporting the activity of enzymes in the MEP pathway. A mildly alkaline pH environment may enhance the solubility and recovery of hydrophobic sesquiterpenes, such as amorpha-4,11-diene, in the culture medium. However, it may negatively affect cell growth and biomass accumulation, limiting the feasibility of achieving the high cell densities typically required for industrial-scale sesquiterpenes production.^112^

### 6.6 Influence of fermentation mode

The mode of fermentation dictates nutrient availability, metabolic activity, and productivity. Among the fermentation modes, the fed-batch process is the most commonly employed mode in industrial processes.^114^ This approach offers controlled nutrient addition, which prevents substrate inhibition while maintaining optimal concentrations of carbon and nitrogen sources. By avoiding nutrient depletion and minimizing by-product accumulation, it enables prolonged production phases and higher yields compared to batch processes.^115^ While continuous fermentation offers steady-state operation and high productivity, its application for artemisinin precursor synthesis is less common due to the complexity of maintaining stable metabolic conditions in engineered systems over extended periods. Batch fermentation, although simpler, is typically less efficient due to rapid nutrient depletion and accumulation of inhibitory by-products, limiting its productivity.^116^ Significant advancements in sesquiterpene production have been accomplished through fed-batch fermentation techniques. For example, the production of artemisinin precursors, such as amorpha-4,11-diene^52^ and artemisinic acid,^57^ has reached titres >40 g/L and 25 g/L, respectively, utilizing highly modified *S. cerevisiae* strains. These investigations employed exponential feeding strategies for glucose/ethanol mixtures and intermittent ethanol supplementation (10 g/L). Feeding algorithms were dynamically controlled and triggered based on stir rate, dissolved oxygen levels, and CO_2_ production rates. Similarly, engineered *E. coli* strains have demonstrated remarkable productivity, achieving amorpha-4,11-diene titers of 27.4 g/L^53^ and 30 g/L^97^ in fed-batch fermentation systems.

### 6.7 Effect of dissolved oxygen

Dissolved oxygen (DO) impacts cellular respiration, energy generation, and the functionality of oxygen-dependent enzymes.^117^ In aerobic circumstances, yeast metabolizes glucose into carbon dioxide and water to generate energy. During yeast cultivation, biomass rapidly grows exponentially, resulting in significant oxygen consumption and a swift decline in DO levels. As glucose concentrations diminish to a lower threshold, yeast cell growth slows due to insufficient carbon availability in the medium and rapidly increasing DO levels. When additional glucose is supplied to the medium, DO levels gradually decrease again as glucose is consumed. This dynamic regulation of DO is crucial for controlling cellular proliferation and product synthesis, particularly in fed-batch fermentation processes.^112^ By maintaining DO at optimal levels, glucose utilization and yeast development rates can be well-regulated. While oxygen is essential for aerobic organisms, excessive levels can result in the formation of ROS, which can harm cellular components and impair metabolic activity. ROS production is exacerbated under stressful conditions, such as elevated oxygen concentrations in the cultivation environment.^118^ As a result, selecting the appropriate DO levels is critical for ensuring cell health, sustaining growth, and optimizing production. In many studies, DO levels during sesquiterpene fermentation are usually maintained at 30 % or 40 % by modulating stirring rates and airflow using cascade control strategies in fed-batch systems (Table 4). This approach supports robust cell performance and enhances the production of sesquiterpenes.

## 7 Conclusions and future prospects

Artemisinin, a sesquiterpene lactone, along with its derivatives like artesunate and artemether, has garnered heightened interest due to their critical role as essential constituent elements in artemisinin-based combination therapies, which are recommended by the WHO as the primary treatment protocols for malaria. This review has underscored the growing significance of SFE in artemisinin extraction from *A. annua* plant. The technology is gaining interest due to its notable attributes, such as high selectivity, efficiency, and shorter extraction times. scCO_2_ has emerged as a viable alternative to traditional solvent-based extraction techniques because it eliminates the need for toxic solvents. In this review, we provided a comprehensive understanding and insight into the impact of various process parameters such as temperature, pressure, CO_2_ flow rate, and solvent modifiers on enhancing the selectivity and optimizing artemisinin yield from *A. annua* for the SFE process. The thorough examination of these parameters has revealed their influence on solvent density and its interactions with solute molecules, which ultimately affect the stability and chemical composition of the final extract. However, the demand for artemisinin-based therapies is increasing, and, thus, the fast development of alternative and sustainable sources rather than single dependence on natural plant sources is required. Microbial fermentation using engineered microbes promises a sustainable, economical, high-yield, and reliable supply to ensure the availability of this critical antimalarial drug. *S. cerevisiae* has been demonstrated as an effective cell factory for the commercial production of sesquiterpenes. Significant achievements include industrial-scale synthesis of artemisinin precursors, such as artemisinic acid and amorpha-4,11-diene, which are subsequently converted into artemisinin through chemical or enzymatic processes. Looking ahead, we anticipate that scCO_2_ extraction technology will continue to experience substantial growth in the coming years. To facilitate artemisinin production through SFE, it is essential to lower the costs associated with the supply of plant material. This can be achieved by developing an integrated agro-industrial system dedicated to producing standardized plant material. Key strategies include optimizing agricultural practices, employing high-yield genotypes of *A. annua* to maximize dry biomass production, and implementing techniques to enhance artemisinin content within the harvested plant material. From the perspective of fermentation strategies, one approach to reducing feedstock costs involves identifying and adapting inexpensive carbon sources, such as agricultural by-products, to be compatible with *S. cerevisiae* metabolism. This aligns with the growing trend of enhancing pharmaceutical artemisinin production through optimized fermentation processes. Optimizing the production process is essential for enhancing productivity and reducing overall costs. This involves fine-tuning the composition of the cultivation medium, adjusting key physicochemical conditions, and employing advanced downstream processing methods to improve efficiency and yield. Although scCO_2_ extraction offers numerous benefits, its application in recovering artemisinin precursors from fermentation processes remains limited. This underutilization highlights a significant opportunity to improve the sustainability and efficiency of producing these essential compounds.

## CRediT authorship contribution statement

**Babatunde Oladipo:** Writing – original draft, Visualization, Methodology, Investigation, Data curation. **Tunde V. Ojumu:** Writing – review & editing, Supervision, Resources, Conceptualization.

## Declaration of competing interest

The authors declare that they have no known competing financial interests or personal relationships that could have appeared to influence the work reported in this paper.
